# 673. An Exploration into the Impact of Paraffin Wax on Burn Scar During Exercise Therapy

**DOI:** 10.1093/jbcr/irag033.554

**Published:** 2026-04-06

**Authors:** Rebekah R Allely, Maybelle E Singson, Bonnie C Carney, Kateri D Rupnarine, Amy Brodsky, Lan Van-Buendia, James Kim, Ronald R Lassiter, Jonathan Niszczak, Jamie G Oh, Jeffrey W Shupp, Shawn Tejiram, Taryn E Travis

**Affiliations:** Medstar Washington Hospital Center, Washington, DC; Medstar Washington Hospital Center, Washington, DC; The Burn Center at MedStar Washington Hospital Center, Washington, DC; Medstar Washington Hospital Center, Washington, DC; Medstar Washington Hospital Center, Washington, DC; Medstar Washington Hospital Center, Washington, DC; Medstar Washington Hospital Center, Washington, DC; Medstar Washington Hospital Center, Washington, DC; Thomas Jefferson University Burn Center / Wacker Silicones, Hatfield, PA; Medstar Washington Hospital Center, Washington, DC; Medstar Washington Hospital Center, Washington, DC; Medstar Washington Hospital Center, Washington, DC; The Burn Center at MedStar Washington Hospital Center, Washington, DC

## Abstract

**Introduction:**

Burn hypertrophic scar is common after burn injury and can be associated with pain, contractures, and functional limitations for burn survivors. Paraffin wax therapy is a non-invasive modality commonly utilized for analgesia and local tissue elasticity for a variety of conditions including treatment of burn scar. Though burn therapists have historically found benefits of paraffin wax therapy on tissue extensibility, especially when used in combination with other rehabilitative therapies, there is limited research exploring its effect on burn scar to support this practice. Additionally, usage of paraffin wax has decreased over recent years across burn centers. This study aims to investigate its effectiveness on range of motion, hypothesizing that paraffin wax as an adjunct to exercise therapy will be associated with improvements in range of motion.

**Methods:**

Adult patients with a burn scar crossing a joint receiving outpatient rehabilitation service at an ABA-verified burn center were eligible for enrollment. This was a prospective, randomized controlled study. Six patients were enrolled, with 3 in the paraffin and 3 in the no-paraffin group. Each patient had 4 treatment sessions (n = 12 total ROM measurements per group). Range of motion at the specified joint was measured before exercise therapy and after exercise therapy by an investigator blinded to the randomization. For those in the paraffin group only, paraffin wax was applied before exercise. A two-way ANOVA with multiple comparisons and Sidak’s correction was used to compare the groups before and after exercise.

Each joint had a total available range of motion (TAROM) calculated from known values. Due to the variable nature of the joint mobility, each joint was normalized as a percentage gained during exercise to the TAROM. For percentage gained, outliers were removed after Rout’s outlier test. An un-paired t-test was used to compare the groups in percentage of range of motion gained during the session.

**Results:**

In the paraffin group, there was a significant increase in degrees of range of motion gained when comparing before and after exercise therapy (69.9 ± 21.1 vs. 81.5 ± 7.8, *p*<.01). This increase was not observed in the no-paraffin group (60.83 ± 14.6 vs. 68.6 ± 15.7, *p*>.05). The paraffin group gained more ROM during their exercise session compared to the no-paraffin group (7.1 ± 7.4 vs. 3.6 ± 1.3%, *p*=.1907).

**Conclusions:**

These findings support the hypothesis that paraffin wax as an adjunct to exercise therapy significantly improves range of motion compared to exercise therapy alone. Additional patients are currently being enrolled to confirm or refute these findings.

**Applicability of Research to Practice:**

Burn therapists should consider paraffin wax as an adjunctive therapy in their plans of care for addressing scar-related range of motions limitations when treating patients with burn hypertrophic scar.

**Funding for the study:**

N/A.

